# A robotic goniometer exchanger for high-throughput single-crystal X-ray diffraction at SPring-8

**DOI:** 10.1107/S1600577526002110

**Published:** 2026-04-02

**Authors:** Yuiga Nakamura, Sumit Ranjan Maity, Toshiyuki Sasaki, Kouhei Ichiyanagi

**Affiliations:** a Japan Synchrotron Radiation Research Institute (JASRI) 1-1-1 Kouto, Sayo-cho Sayo-gun Hyôgo679-5198 Japan; ESRF – The European Synchrotron, France

**Keywords:** single-crystal X-ray diffraction, robotic-arm dif­frac­tom­eter, synchrotron instrumentation, high-energy X-rays, automation

## Abstract

Rapid and reliable sample handling is essential for high-throughput single-crystal diffraction at modern synchrotron facilities. At the high-energy single-crystal X-ray diffraction beamline BL02B1 of SPring-8, a compact six-axis robotic arm was implemented as a dif­frac­tom­eter for single-crystal measurements using high-energy X-rays. Coordinated six-axis motion enables virtual rotation about an arbitrary axis and reproduction of a conventional ω-scan geometry without additional rotary stages. Under identical beam conditions (180 µm × 113 µm), the robotic-arm system yielded diffraction data with an internal agreement factor of *R*_int_ = 0.117, comparable with that obtained using a conventional goniometer (*R*_int_ = 0.134). Optical microscopy and diffraction analysis indicate a positional deviation of approximately ±17 µm during ω-scans, with a standard deviation of the spindle position of 0.14°. Although this accuracy is lower than that of high-precision single-crystal goniometers, it is sufficient for diffraction experiments employing large beam sizes and high-energy X-rays. Owing to its compact design, programmable motion control and open geometry, the system provides a flexible platform for automated sample handling and high-throughput diffraction experiments in support of the SPring-8-II upgrade.

## Abstract 2

A compact six-axis robotic arm has been implemented at the BL02B1 beamline of SPring-8 as a high-precision goniometer for single-crystal diffraction. Its programmable control enables automated sample handling and centring, advancing autonomous high-throughput diffraction in preparation for SPring-8-II.

## 1. Introduction

Single-crystal X-ray diffraction (XRD) is indispensable for elucidating the atomic and electronic structures of crystalline materials. When combined with synchrotron radiation, it enables measurements on microcrystals using intense short-wavelength X-rays and allows structural analyses with sub-angstrom precision and electron-density resolution. Consequently, synchrotron-based single-crystal XRD has become a key technique for both fundamental crystallography and functional-materials research.

The demand for high-throughput single-crystal measurements has increased rapidly with the growth of studies that require sequential data collection, such as temperature-dependent experiments, compositional series and *in situ* or time-resolved measurements. Although modern detectors and high-flux synchrotron sources enable rapid data acquisition, overall experimental efficiency remains partially constrained by manual sample alignment and goniometer operation inside the experimental hutch. Each manual adjustment interrupts measurement continuity and limits the ability to exploit the high flux and stability of advanced synchrotron sources. Developing compact, precise and automated dif­frac­tom­eters is therefore essential for achieving true high-throughput operation.

At beamline BL02B1 of SPring-8, such automation has become particularly relevant. BL02B1, originally developed for high-energy single-crystal diffraction and advanced structural analysis (Sugimoto *et al.*, 2010 ▸), delivers high-energy X-rays (30–50 keV) for a broad range of materials – from inorganic oxides and halides (Maity & Nakamura, 2025 ▸) to molecular and metal–organic crystals (Nakamura *et al.*, 2025 ▸) and metal–organic frameworks – supporting research that has contributed to recent Nobel Prize-winning achievements (Kitagawa, 2025 ▸). Both precision and throughput are critical for such studies. The beamline routinely supports charge-density and advanced structural analyses (Kitou *et al.*, 2023 ▸), which often require measurements on multiple samples or under multiple conditions. The combination of high flux, diverse research targets and increasing data volume underscores the need for a goniometer system capable of rapid, reproducible and potentially automated operation to shorten measurement cycles and improve experimental efficiency.

To address this need, we have developed a robotic-arm dif­frac­tom­eter for single-crystal experiments at BL02B1. Although several studies have reported the use of robotic arms in XRD measurements, most of them were limited to applications as sample changers (Murakami *et al.*, 2020 ▸; Lazo *et al.*, 2021 ▸). Our system employs a six-axis compact industrial robotic arm that functions as a high-precision goniometer, with potential to also serve as an automated sample exchanger and perform automatic optical centring through programmable motion control. Compared with conventional spindle-based goniometers, the robotic arm provides additional rotational degrees of freedom, customizable motion paths and flexible access geometry, while maintaining angular precision comparable to conventional instruments. Its compact design also facilitates integration with various sample environments, such as cryostreams and hot-air blowers, making it suitable for *in situ* and time-resolved studies.

Here, we present a proof-of-principle demonstration of this robotic-arm dif­frac­tom­eter at BL02B1. The results confirm that the system achieves goniometric precision equivalent to existing setups, while offering enhanced flexibility and extensibility. This development represents an important step toward automated and high-throughput single-crystal diffraction in the upcoming SPring-8-II era.

## 2. Experimental setups

**Figure 1:**
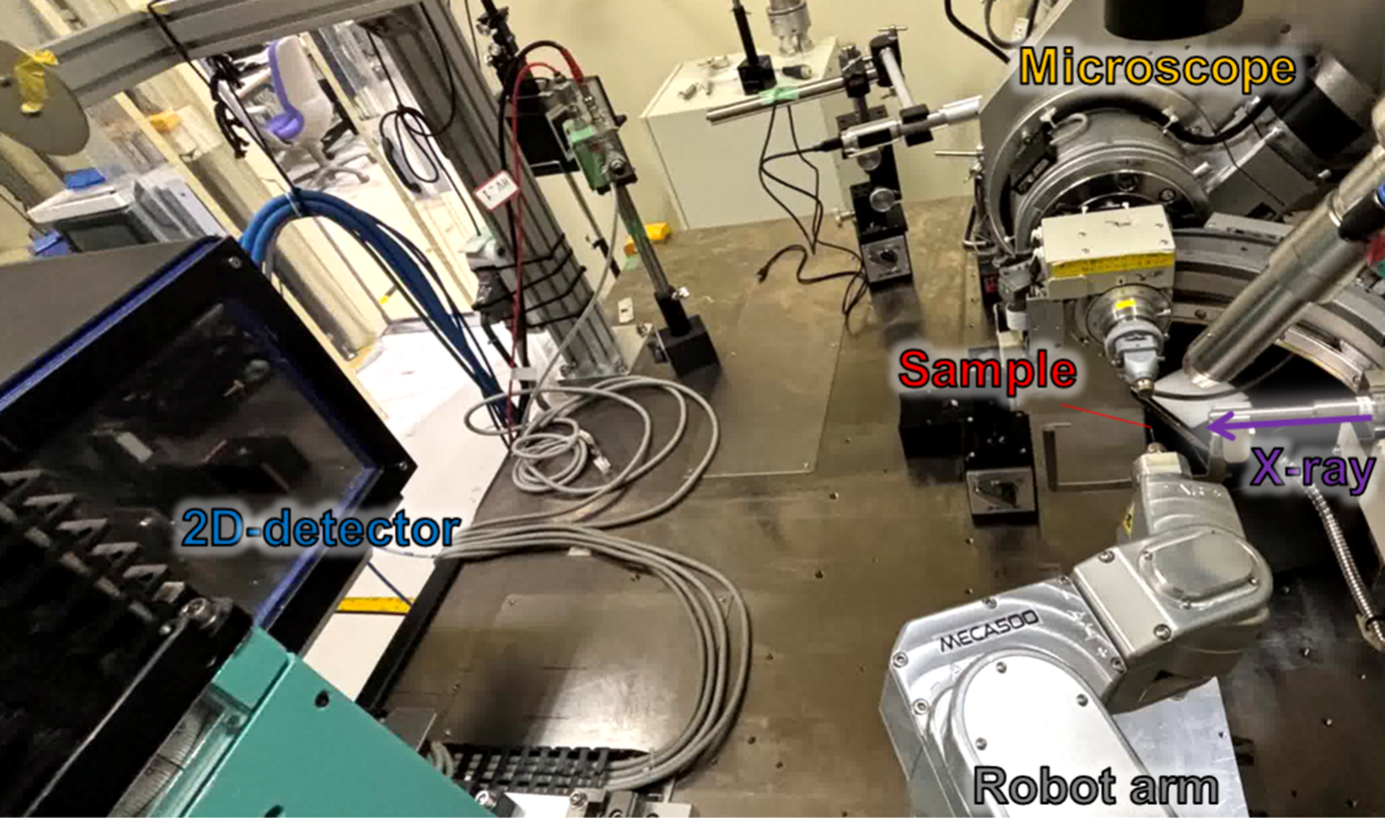
Experimental setup at beamline BL02B1 of SPring-8. The robotic arm (MECA500, Mecademic) was installed opposite the conventional dif­frac­tom­eter to allow comparative measurements under identical experimental conditions.

A compact six-axis robotic arm (MECA500, Mecademic) (Cowden *et al.*, 2019 ▸) was installed at the single-crystal diffraction beamline BL02B1 of the SPring-8 synchrotron facility. The MECA500 is particularly suited for synchrotron applications. Its ultra-compact design (330 mm reach, 0.5 kg payload, total weight < 5 kg) and rigid aluminium body allow installation within the confined space around the beam centre, while maintaining high mechanical stability. It can accommodate an additional load of approximately 0.5 kg, allowing the installation of user-specified small sample environments or goniometers – such as gas-flow systems, pressure cells or electric/magnetic devices – for various experimental studies. Furthermore, the system can be controlled *via* an Ethernet connection and Python scripting. A six-axis coordinated control mode enables the simultaneous operation of all six axes, allowing the sample to be virtually rotated about an arbitrary axis. This represents the most significant difference from earlier studies, in which a high-precision small rotary axis was mounted at the tip of a large robotic arm (Nurizzo *et al.*, 2016 ▸). This functionality was also utilized to reproduce the ω-scan axis used in the present study. As shown in Fig. 1 ▸, the robotic arm was positioned directly opposite the existing spindle-type goniometer to enable comparative measurements under identical beam conditions. This configuration preserved the standard beamline setup, including the crystal-viewing camera, beam stopper and optical alignment system, without requiring any modification to the infrastructure.

A two-dimensional detector PILATUS3 X CdTe 1M (Dectris) was mounted downstream of the beam to record diffraction images from both the robotic and conventional goniometers. Due to spatial constraints introduced by the comparative setup, the sample–detector distance was set to 669.6 mm, longer than the standard 130 mm configuration at BL02B1, to maintain an unobstructed scattering geometry.

For the demonstration, a single crystal of Nd_1.5_Sr_0.5_NiO_4_ (Wahyudi *et al.*, 2015 ▸) was mounted on the robotic arm using a magnetic pin holder. The measurement was performed at room temperature. High-energy X-rays (40 keV, λ = 0.310 Å) were employed to access a broad region of reciprocal space and to mitigate the resolution limitations imposed by the experimental layout. The beam had a full width at half-maximum (FWHM) of 180 µm vertically and 113 µm horizontally. The ω-axis was rotated through 90° at a rate of 2.5° s^−1^, yielding 180 diffraction images for both cases. Distinct diffraction peaks were observed up to the (114) reflection (*d* ≃ 2.03 Å), confirming that the robotic-arm goniometer provides stable orientation control and reliable diffraction data collection under standard BL02B1 operating conditions. The robot’s compact form factor and high precision demonstrate its potential for integration into automated high-throughput diffraction workflows at SPring-8.

## 3. Results

**Figure 2:**
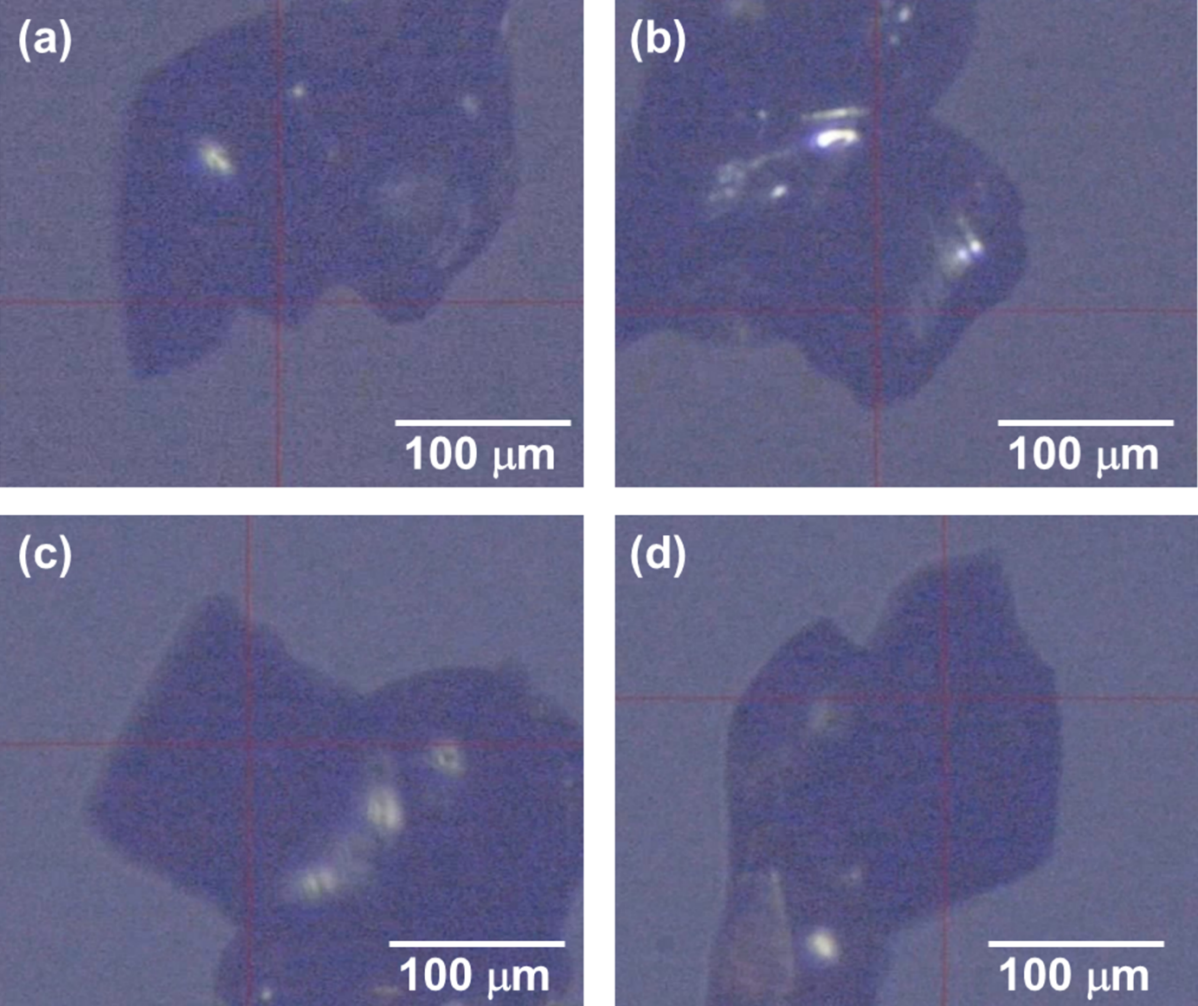
Crystal images obtained with the on-axis microscope of the dif­frac­tom­eter: (*a*)/(*b*) the robotic-arm dif­frac­tom­eter at ω = 0 and 90°, and (*c*)/(*d*) the conventional dif­frac­tom­eter at ω = 0 and 90°.

Diffraction data were successfully collected using both instruments (Movies S1 and S2 of the supporting information). The robotic-arm dif­frac­tom­eter yielded 15 reflections (57.1% completeness), whereas the conventional goniometer pro­duced 11 reflections (46.2% completeness). All reflections were correctly indexed, confirming the geometric accuracy of the robotic-arm setup. A slight positional fluctuation of the sample, roughly estimated to be ∼30 µm, was observed for the robotic-arm measurement (Fig. 2 ▸ and Movies S3 and S4), but this fluctuation did not noticeably affect the overall data quality.

To quantitatively evaluate this fluctuation, the diffraction images were processed using *CrysAlis PRO* (Rigaku OD, 2025 ▸), and the *R*_int_ value was employed as an indicator of internal consistency among equivalent reflections. As shown in Fig. 2 ▸, the crystal size was comparable to the beam diameter. If the positional stability of the dif­frac­tom­eter were insufficient, the diffraction intensity would decrease significantly during the scan, leading to an increased *R*_int_ value due to reduced agreement among symmetry-related intensities. Nevertheless, comparable results were obtained: *R*_int_ = 0.134 for the conventional goniometer and *R*_int_ = 0.117 for the robotic-arm dif­frac­tom­eter. The difference lies within experimental uncertainty. While the *R*_int_ values were obtained from a limited set of reflections because of constraints inherent to the experimental layout, they are sufficient to confirm that the X-ray beam did not move off the sample during the scan. In addition, we analyzed our diffraction data using the *XDS* program (Kabsch, 2010 ▸) and calculated the ‘standard deviation of the spindle position’, which was found to be 0.14° for both the robotic-arm and the conventional dif­frac­tom­eters. The standard deviation of the spindle position corresponds to the deviation from the nominal rotation angle at each frame and these results suggest that the robotic-arm dif­frac­tom­eter has rotational speed stability comparable to that of a conventional dif­frac­tom­eter.

For more detailed verification, we mounted a needle at the tip of the robotic arm and quantitatively evaluated the eccentricity by tracking the position of the needle tip. For more detailed verification, an additional microscope was installed downstream of the beam, enabling simultaneous evaluation of the vertical component of the eccentricity (Fig. S1). The results obtained from the top-view and side-view microscopes at ω = 0, 90 and 180° are presented (Fig. S2 and Movie S5). The eccentricity determined using the microscope positioned above the sample was ±17 µm, which closely matches the value obtained from the downstream microscope. These observations indicate that the positional deviation of the robotic arm tip is approximately ±17 µm under the present experimental conditions. These findings demonstrate that the robotic-arm dif­frac­tom­eter achieves rotational precision and mechanical stability equivalent to a conventional single-crystal goniometer, validating its feasibility for high-energy diffraction experiments. Further refinement, such as the integration of a dedicated motorized rotation stage for the ω-axis, could further enhance angular accuracy. Even in this configuration, unlike the previously reported dif­frac­tom­eter (Nurizzo *et al.*, 2016 ▸), the relative orientation between the sample and the scan axis can be controlled, enabling greater flexibility in diffraction measurements. Owing to its lightweight (∼5 kg) and modular design, such improvements and extensions toward automated sample exchange and centring can be readily implemented, paving the way for future high-throughput and autonomous diffraction measurements at SPring-8.

## 4. Conclusion

A proof-of-principle demonstration of a robotic-arm dif­frac­tom­eter has been successfully conducted at the high-energy single-crystal beamline BL02B1 of SPring-8. For applications employing X-ray beams large enough to exceed the characteristic positional deviation, the system achieves mechanical precision comparable to that of a conventional goniometer and exhibits stable diffraction performance under standard beamline conditions. Once fully implemented, this approach is expected to enable high-throughput measurements with improved space efficiency and cost effectiveness. In particular, this robotic arm could potentially be extended to function as a sample exchanger in the future, as illustrated by the initial conceptual design of the sample-exchange mechanism shown in Fig. S3. The design provides a wider χ–φ rotation range and can be extended to other X-ray techniques, such as thin-film diffraction or inelastic scattering. Continued development of this compact and flexible robotic platform will contribute to autonomous and efficient synchrotron experiments in the SPring-8-II era.

## Supplementary Material

Movie 1. DOI: 10.1107/S1600577526002110/ret5001sup1.mp4

Movie 2. DOI: 10.1107/S1600577526002110/ret5001sup2.mp4

Movie 3. DOI: 10.1107/S1600577526002110/ret5001sup3.mp4

Movie 4. DOI: 10.1107/S1600577526002110/ret5001sup4.mp4

Movie 5. DOI: 10.1107/S1600577526002110/ret5001sup5.mp4

Supporting infomation. DOI: 10.1107/S1600577526002110/ret5001sup6.pdf

## Data Availability

Movies S1 and S2 show the diffraction patterns recorded during measurements with the robotic-arm dif­frac­tom­eter and the conventional goniometer, respectively. Movies S3 and S4 show microscopic views of the crystals being rotated by the robotic-arm dif­frac­tom­eter and the conventional goniometer, respectively. Movie S5 shows microscopic views of the needle tip mounted on the robotic arm during rotation.
