# Supplementary material for: A robotic goniometer exchanger for high-throughput single-crystal X-ray diffraction at SPring-8
Source: J Synchrotron Radiat. 2026 Apr 2;33(Pt 3):825–8. doi: 10.1107/S1600577526002110 (PMC13148606; doi:10.1107/S1600577526002110)
Supplement: Supplementary file 6 [file s-33-00825-sup6.pdf]

JOURNAL OF SYNCHROTRON RADIATION

Volume 33 (2026)

Supporting information for article:

A robotic goniometer exchanger for high-throughput single-crystal X-ray diffraction at SPring-8

Yuiga Nakamura, Sumit Ranjan Maity, Toshiyuki Sasaki and Kouhei Ichiyanagi

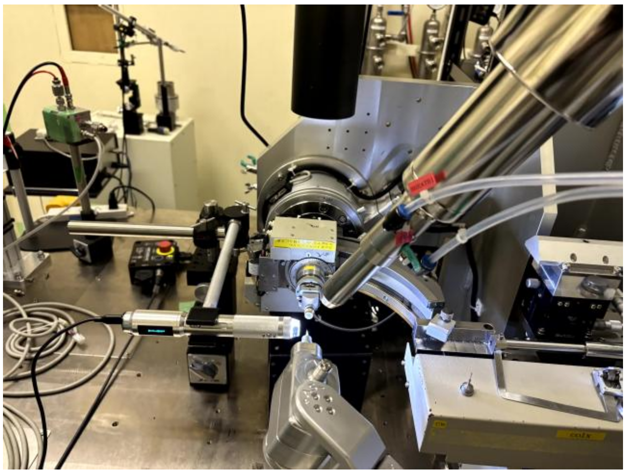

Figure S1 Photograph of the robotic-arm diffractometer showing the microscope installed downstream of the X-ray beam for observation of the needle motion.

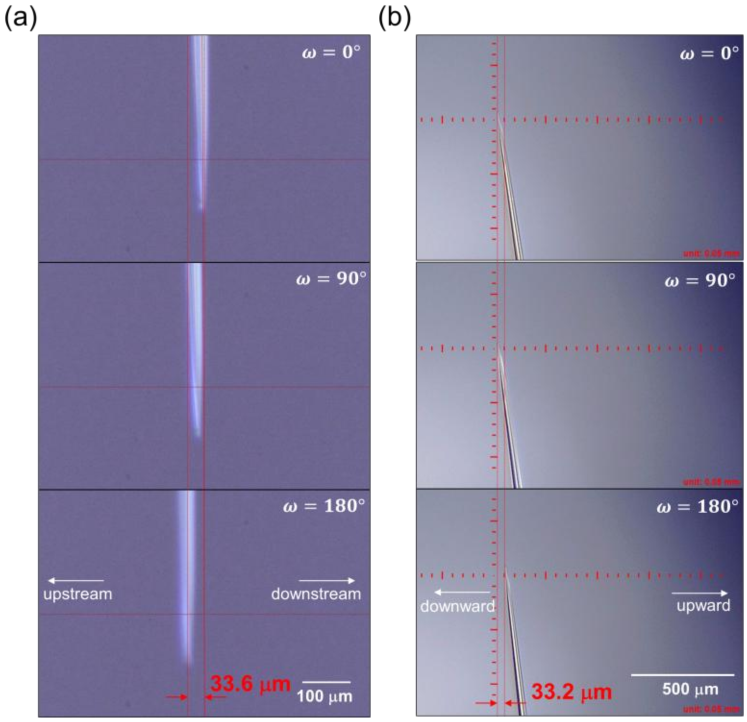

Figure S2 Microscope images of the needle at ω = 0°, 90° and 180°: (a) top view; (b) side view from the downstream direction of the X-ray beam.

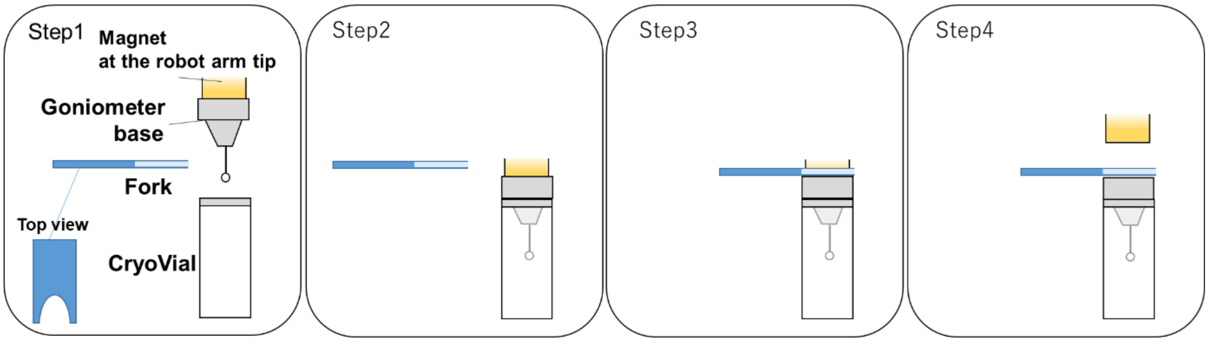

Figure S3 Schematic illustration of the initial conceptual design of the sample-exchange mechanism.
